# The Refugial Squeeze: A Globally Relevant Mechanism of Species Decline Revealed by a Threatened Amphibian

**DOI:** 10.1002/ece3.74417

**Published:** 2026-10-15

**Authors:** Kate Tunstill, Matt W. Hayward, Hannah Power, Darren Southwell, John Gould, Will Farebrother, Michael Mahony, Alex Callen

**Affiliations:** ^1^ School of Environmental and Life Sciences The University of Newcastle Callaghan New South Wales Australia; ^2^ Centre for African Conservation Ecology Nelson Mandela University Port Elizabeth South Africa

**Keywords:** chytrid fungus, climate change, coastal wetlands, range contraction, *Ranoidea aurea*, spatial relationships

## Abstract

A key outcome of the Global Biodiversity Crisis is the contraction and range shift of species distribution into refugial habitats that afford protection against threatening processes. Range contractions driven by spatially overlapping threats may fragment populations to the point where functional habitat is lost. This study identifies a range contraction of the Vulnerable green and golden bell frog ( *Ranoidea aurea* ) in south‐east Australia using a multi‐method approach. We fitted alpha hulls, species distribution models (MaxEnt, Random Forest, Boosted Regression Trees and an AUC‐weighted ensemble model) and conducted a hypervolume analysis to quantify change in the species' range and niche between full (historical) and contemporary periods. Our findings suggest *R. aurea* has undergone niche contraction *and* displacement with a directional shift into coastal saline‐influenced refugia. Our results demonstrated a 76% reduction in extent of occurrence, a suitable habitat reduction of ~96%, and an environmental niche contraction of 57%. The remaining habitat was strongly associated with coastal proximity with shifts towards more saline environments. The concentration of *R. aurea* in coastal regions suggests intensified dependence on coastal refugia, persisting in salt‐exposed habitat that may suppress chytrid fungus. Yet, the threat abatement afforded by low‐lying, saline coastal environments is wedging *R. aurea* into chytrid refugia that is at risk from sea level rise. Refugial squeeze is a globally relevant phenomenon likely affecting a suite of coastal species that highlights the value of integrating geometric and probabilistic niche approaches with species distribution models to resolve temporal contraction in species distributions. Protection of refugial squeeze zones from sea level rise will ultimately determine the future range and population viability of coastal species.

## Graphical abstract

An Ensemble species distribution model (SDM) was used to compare historical and contemporary distributions of the threatened green and golden bell frog ( *Ranoidea aurea* ) in coastal NSW, Australia. Results showed a 76% reduction in extent of occurrence, a suitable habitat reduction of ~96% between historical and contemporary periods, and an environmental niche contraction of 57%. Findings highlight the value of ensemble SDMs for detecting fine‐scale habitat shifts in declining amphibian populations and informing targeted conservation of remaining coastal refugia.

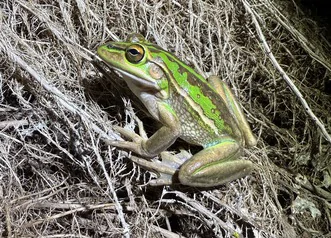

## 1 Introduction

Species distributions are contracting into refugial habitats that afford protection against specific threatening processes (Reside et al. 2019). Yet, these contractions can be spatially complex if species are exposed to multiple threats, where one threat drives a distribution in one direction while another threat drives it in the opposing direction. This may cause a species' habitat to be lost or wedge species into increasingly small and isolated habitat patches. For example, the Sonoyta mud turtle ( *Kinosternon sonoriense longifemorale* ) in the Sonoran Desert naturally retreats to isolated, perennial pools in streams to survive drought conditions, which have become exacerbated by human‐driven climate change (Rosen et al. 2010). However, these few remaining pools are often invaded by non‐native species, like the American bullfrog (*Aquarana catesbeiana*), which outcompete or prey upon the turtles, trapping them in a sink habitat that is both shrinking and becoming more dangerous (Rosen et al. 2010). Similarly, forest fragmentation has forced Australia's critical weight range mammals to contract to isolated patches that are increasingly threatened by introduced predators because of higher predation success in edge habitats (May and Norton 1996). This is, in essence, the double‐jeopardy of refugial squeeze, where shrinking refugial habitat of a threatened species is itself vulnerable to a different but novel set of threats. This makes clarifying anthropogenic processes driving distributional changes critical to identify risks early and inform conservation strategies. The drivers of this refugial contraction are often difficult to establish; however, since commonly accessible abiotic factors (climate, vegetation type, salinity) can affect biotic factors (disease, predation, competition) it is therefore critical to identify these drivers in a model‐accessible form. The fungal pathogen, *Batrachochytrium dendrobatidis*, resulting in the deadly disease *Chytridiomycosis* (hereafter chytrid), is strongly influenced by abiotic conditions, providing a traceable proxy for contemporary refugial drivers and boundaries.

The IUCN Red List recently ranked amphibians as the most threatened vertebrate class—the second time since 2019, citing habitat loss and degradation, as well as disease, driving most species to extinction (Luedtke et al. 2023). This global vulnerability is the result of biphasic life cycles being the most common reproductive strategy for amphibians, making them disproportionately more sensitive to multi‐stressor landscapes (Green 2003; Nolan et al. 2023). However, while habitat loss and degradation fragment populations that drive demographic collapse over decades (Luedtke et al. 2023), landscape persistent diseases such as that caused by *Batrachochytrium dendrobatidis*, typically result in a quicker trajectory to extinction (months to years) (Scheele et al. 2019). Although global analyses have failed to document directional niche contractions for amphibians resulting from chytrid‐driven declines, Scheele et al. (2023) demonstrated niche shifts of frogs into warmer, wetter and low‐elevation refugia in Australia. In these areas, host resilience or lower *Batrachochytrium dendrobatidis* pathogenicity allows persistence (Scheele et al. 2023), due to a mismatch in the resource requirements for frogs versus the persistence of the fungus (Duncan et al. 2025). Similarly, there is also evidence of amphibian declines associated with *Batrachochytrium dendrobatidis* causing contraction towards core refugial populations rather than progressively towards range margins (Scheele et al. 2025). While species may theoretically remain viable with such contractions or shifts in the absence of other threats, the concerning IUCN Red List ranking has acknowledged the role that anthropogenic climate change would play in amphibians becoming ‘climate captives’, constrained to remaining habitat now also subject to extreme weather cycles (Luedtke et al. 2023). Thus, amphibians face the risk of non‐viable refugial squeeze towards low‐elevation refugia that itself is now threatened by sea‐level rise, while other anthropogenic climate change induced weather extremes may amplify disease‐driven squeezes globally.

The documented contraction of the threatened green and golden bell frog ( *Ranoidea aurea* ) to the New South Wales coastline in south‐eastern Australia may serve as a model case of refugial squeeze. Disease‐driven population decline has resulted in an estimated 90% range contraction (Daly 1996; Hamer 2002; Mahony 1996), constraining its historic range that spread as far as 200–300 km inland from the coast, to approximately 40 disjunct populations < 50 km from the coast (Mahony et al. 2013). Coastal salinity is postulated to inhibit *Batrachochytrium dendrobatidis*, enabling host persistence, with coastal populations showing elevated salinity tolerance (Stockwell et al. 2015). *Ranoidea aurea* is now largely confined to coastal wetlands (Mahony et al. 2013), which encompass a broad spectrum of hydrological conditions, from freshwater, estuarine and marine environments with permanent and ephemeral inundation regimes (Stockwell et al. 2015). However, anthropogenic climate change‐induced weather extremes may threaten the lowland coastal wetland refugia of *R. aurea* through sea‐level rise inundation, increasing salinity exposure beyond tolerance levels and altering timing of wetland ephemerality, both of which result in phenological mismatch and increased coastal squeeze (Doody 2004; Pontee 2013).

Despite the significant range contraction of *R. aurea* being well documented (Daly 1996; Hamer 2002; Mahony 1996), it has never been quantified through spatial and environmental modelling techniques. Without such modelling, forecasting the climate and disease interactions called for in the most recent IUCN Global Amphibian Assessment (Luedtke et al. 2023) cannot reliably occur. Determining the processes driving distributional change is critical to identifying risks early to maximise the chance of conservation success. Currently the IUCN Red List assessments traditionally uses minimum convex polygons, which often overestimate a species distribution due to the polygon drawing method (Breiner et al. 2017; Burgman and Fox 2003; Duan et al. 2022; IUCN 2015). However, single‐model approaches such as convex polygons, extent of occurrence or area of occurrence are likely to fail threatened species facing refugial squeeze if they cannot capture multi‐stressor interactions where primary threats (e.g., *Batrachochytrium dendrobatidis* filtering) create refugia that is vulnerable to secondary threats (e.g., sea‐level rise).

Combining models to achieve appropriate representation of multi‐stressor interactions is a well‐established approach in species distribution modelling within the last decade (Geyle et al. 2025; Von Takach et al. 2020). Geometric approaches (e.g., alpha‐hulls) delineate spatial contraction but do not identify environmental drivers (Burgman and Fox 2003; Mammola and Cardoso 2020; Meyer et al. 2017), while probabilistic species distribution models characterise environmental suitability but often miss spatial geometry and multi‐stressor histories (Guillera‐Arroita et al. 2015; Radosavljevic and Anderson 2014). Multidimensional hypervolume analyses provide a third line of inference by quantifying shifts in realised niche space and identifying the environmental conditions associated with decline or persistence (Blonder et al. 2018; Von Takach et al. 2020). By integrating all of these techniques, it may be possible to effectively assess all elements of refugial squeeze: contraction into refugia, identification of drivers and projections of persistence under emerging threats.

In this study, we address this critical gap using integrated multi‐method modelling to quantify the historical contraction of *R. aurea* and robustly test the refugial squeeze hypothesis using three linked elements: (1) contraction into specific refugia, (2) identification of the environmental drivers of that contraction, and (3) predictions of persistence under new threats. Triangulating these elements moves beyond identifying generic decline and predicts contractions into refugia that will be suboptimal in the future due to weather extremes resulting from human‐induced climate change. This is the double jeopardy of the refugial squeeze.

## 2 Methods

### 2.1 Occurrence Records

Occurrence records of *R. aurea* (*n* = 2578 raw) were obtained from the Atlas of Living Australia (doi: 10.26197/ala.[b3b0acce‐3524‐48c1‐9e93‐8499d0c65ff8]; accessed 2025‐06‐10) and NSW BioNet (doi: 10.15468/14jd9g[Atlas_records_4j3aekpcbbba1uxhsdubelze20251208‐072704]; accessed 2025‐12‐08), including FrogID, NatureMapr and iNaturalist Australia Contributions. Records pre‐1970 (limited accuracy), > 1000 m uncertainty, non‐visual observations and extralimital outliers (*n* = 2) were excluded. Duplicate records were removed. Two data sets were created: the full‐period (historical) species dataset (1970–2024, 54 years) and the contemporary dataset (2000–2024, 24 years). These periods were chosen to provide complete historical coverage for the species and use enough records to reduce bias and replicate previously successful methods (Geyle et al. 2025; Scheele et al. 2023). The full‐period dataset (herein the full period) included the speculated chytrid emergence time period (1970s; Skerratt et al. 2007) through to 2024. This encompassed the onset of decline noted in the northern region of New South Wales during the 1980's (Scheele et al. 2023) and the period of decline post‐chytrid arrival (herein the contemporary period) to detect potential drivers of niche changes. Data was reprojected into GDA2020 NSW Lambert (EPSG:8058), with one occurrence record per cell (100 m) to reduce spatial bias, resulting in 1516 occurrence records for the full‐period and 1062 records for the contemporary period (Figure 1).

**FIGURE 1:**
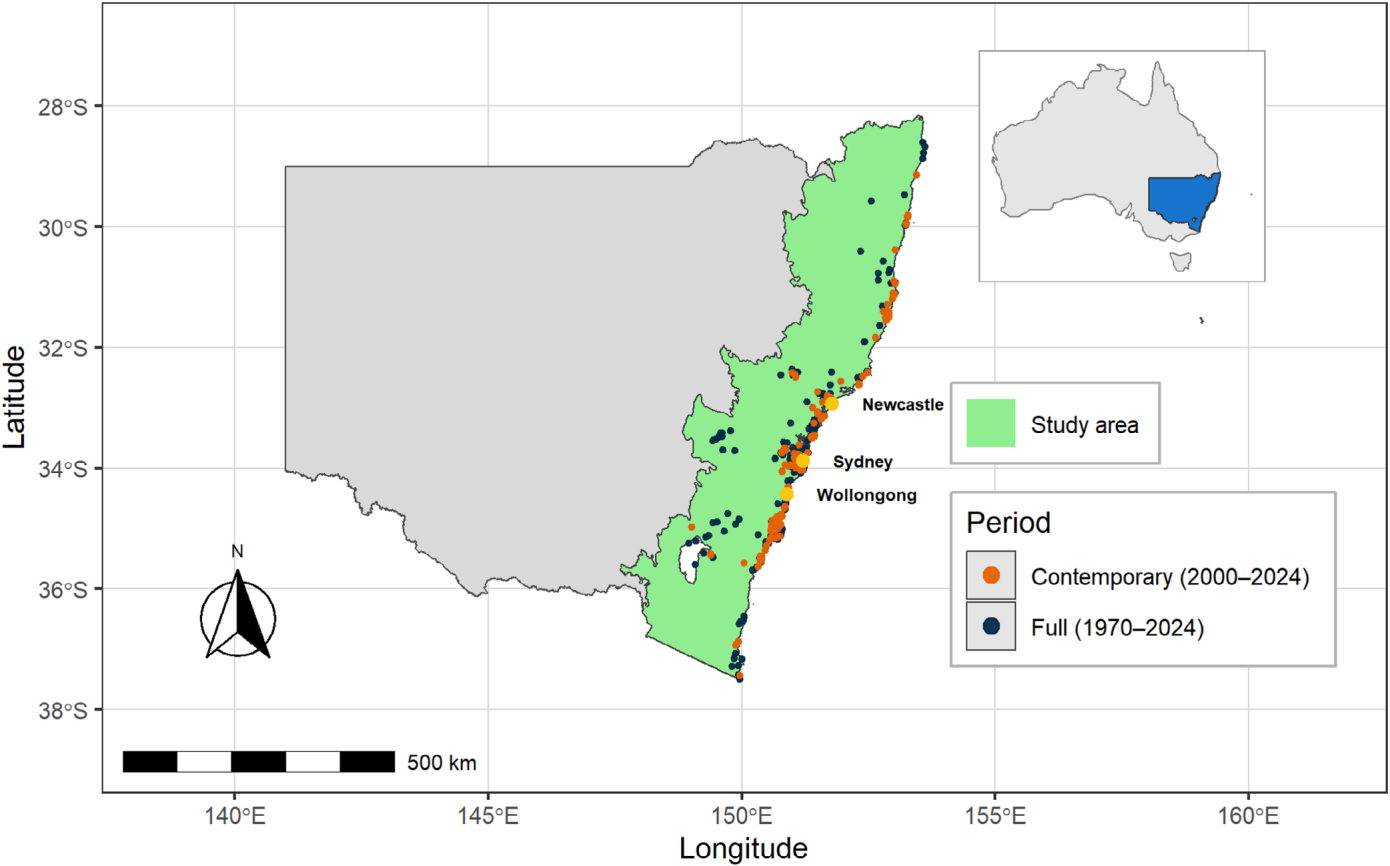
Study area maps showing full (1970–2024) and contemporary (2000–2024) distribution (occurrences) of the green and golden bell frog ( *R. aurea* ) located in New South Wales, Australia. Green is the chosen boundary for the models. Major cities in the state of New South Wales are labelled and placed in yellow.

### 2.2 Extent of Occurrence Using Alpha‐Hulls

An initial buffer analysis of *R. aurea* records in ArcGIS Pro 3.5.0 (ESRI 2025, Redlands, CA) determined the percent of records within 1 km of the coastline to establish the significance of the distance to coast parameter. Extent of Occurrence was calculated using R version 4.5.1 (R Core Team 2025, *alphahull* and *lwgeom* package). To improve extent of occurrence estimates, alpha hulls were used to avoid issues with traditional hull techniques such as convex hulls, which often result in overestimation (Meyer et al. 2017). The use of this technique allows the exclusion of ocean and estuary systems, which increases the accuracy of extent in coastal species such as *R. aurea* . Due to the species elongated coastal distribution, the initial value of α = 2 was used as this is the standard within literature (Breiner et al. 2017; IUCN 2015; Marsh et al. 2023; Scheele et al. 2023), but incomplete hulls were produced (i.e., not fully enclosed). To determine the best alpha value for the occurrences, multiple alpha values were run (α = 2, 4, 6, 8, 10) and compared visually to see which hulls best represented the full‐period and contemporary range of the occurrence records without excluding a significant number of occurrences (Figure S1), resulting in the use of alpha‐hull (α = 8) (Capinha and Pateiro‐López 2014; Meyer et al. 2017; Pateiro‐López and Rodriguez‐Casal 2010). The full‐period hull included occupancy from both time periods so that absolute and percent of range change could be calculated.

### 2.3 Environmental Variables

Environmental and climatic variables were selected from existing literature on the biology and ecology of *R. aureaw* , with variance inflation factors (VIF) used to assess multicollinearity (Table 1, Figure S2, Table S1). Climatic variables from SILO (www.longpaddock.qld.gov.au/silo) were averaged over the chosen time periods (1970–2024, 2000–2024) using .nc files converted to rasters in ArcGIS Pro 3.5.0. Raster tiles for Digital Earth Australia (DEA) Landcover (1988–2024) were downloaded and modal pixel values extracted for each time period; all other variables remained fixed across periods. Layers were reprojected to EPSG:8058 (GDA2020/NSW Lambert), resampled to 100 m resolution, and clipped to Interim Biogeographic Regionalisation for Australia (IBRA) bioregions encompassing all *R. aurea* records (1970–2024).

**TABLE 1:** Chosen data for initial model analysis, indicating the name and type of variable, any additional transformations to data, data source and relevance to study species.

Type	Variable	Transformations	Justification
Climatic	Relative humidity—Max and Min (1970–2024, 2000–2024) https://www.longpaddock.qld.gov.au/silo/	Averaged between years 5 km resolution to 100 m resolution	Microhabitat driver (White and Pyke 2015) Climate proxy for species distribution models (Chen 2013; Han et al. 2024)
	Rainfall—Total https://www.longpaddock.qld.gov.au/silo/	Averaged between years 5 km resolution to 100 m resolution	Key predictor in species distribution models (Girardello et al. 2010; Uribe‐Rivera et al. 2017) Dictates breeding in *R. aurea* (Goldingay and Newell 2005; Hamer et al. 2008)
	Temperature—Min and Max (1970–2024, 2000–2024) https://www.longpaddock.qld.gov.au/silo/	Averaged between years 5 km resolution to 100 m resolution	Common in amphibian species distribution models (Pineda and Lobo 2009; Rödder and Lötters 2010) Affects growth and development in juvenile *R. aurea* (Browne and Edwards 2003; White and Pyke 2015).
	Evaporation— (1970–2024, 2000–2024) https://www.longpaddock.qld.gov.au/silo/	Averaged between years 5 km resolution to 100 m resolution	Affects longevity of breeding habitats (Christy and Dickman 2002; Hamer et al. 2002; Titon and Gomes 2015)
	Topographic Wetness Index (TWI)	Calculated using ArcGIS tools formula: (TWI = ln(tan(Slope)Flow Accumulation + 1))	Predicts presence of amphibians in wetlands (Blank and Blaustein 2012)
Landform	Slope	Calculated using elevation	Affects presence of anuran species (Menin et al. 2007; Ribeiro et al. 2018)
	Aspect	Calculated using elevation	Dictate ectotherm habitat preference (Feldmeier et al. 2020; Moore et al. 2004)
	Elevation (mAHD) https://datasets.seed.nsw.gov.au/dataset/modified‐digital‐elevation‐layer	1 s res—30 m resolution resampled to 100 m resolution	Commonly used in amphibian species distribution models (Chettri and Acharya 2020; Pineda and Lobo 2009; Scheele et al. 2023)
Soil parameter	Soil type https://datasets.seed.nsw.gov.au/dataset/great‐soil‐group‐gsg‐soil‐type‐map‐of‐nsw	Transformed to category via numbers 100 m resolution	Influences microhabitats of amphibian species and their survival and dispersal (Haggerty et al. 2019; Sparling et al. 1995; Watling 2005; Wyman 1988)
	Hydrological soil group https://datasets.seed.nsw.gov.au/dataset/hydrologic‐groups‐of‐soils‐in‐nsw7f9e8	Transformed to category via numbers 100 m resolution	
	Soil salinity https://esoil.io/TERNLandscapes/Public/Pages/SoilDataFederator/SoilDataFederatorHelp.html	Kriging tool combined with existing soil samples (from TERN) and soil type (from SEED) 100 m resolution	
	Soil water pH https://data.csiro.au/collection/csiro:56703?q=TERN%20Soil%20PH&_st=keyword&_str=8&_si=1	90 m resolution to 100 m resolution	
Vegetation	Native vegetation https://www.dcceew.gov.au/environment/land/native‐vegetation/national‐vegetation‐information‐system	5 m resolution to 100 m resolution	Strongly linked to amphibian distribution (Burrow and Maerz 2022; Cline and Hunter 2014; Cortés‐Gómez et al. 2013; Fardell et al. 2018; Hamer et al. 2002; Pulsford et al. 2017; Pyke et al. 2002) Urbanisation fragments habitat, reducing amphibian distribution (Cayuela et al. 2015; Hamer and Parris 2011; Houlahan et al. 2006; Parris 2006; Smallbone et al. 2011)
	Current vegetation https://datasets.seed.nsw.gov.au/dataset/nsw‐state‐vegetation‐type‐map	Vector to raster conversion 5 m resolution to 100 m resolution	
Landform/Land use	Land cover (1988 ‐ 1999, 2000 ‐ 2024) http://dea‐public‐data.s3‐website‐ap‐southeast‐2.amazonaws.com/?prefix=derivative/ga_ls_landcover_class_cyear_3/	Yearly grids taken and Mode extracted for each period of time 1988–1999 and 2000–2024 30 m resolution to 100 m resolution	
	Land use https://datasets.seed.nsw.gov.au/dataset/nsw‐landuse‐2017‐v1p5‐f0ed‐clone‐a95d	100 m resolution	
Proximity	Distance to built areas https://ecat.ga.gov.au/geonetwork/srv/api/records/0508a90a‐f048‐460a‐9bca‐4f7f437274d0	Distance accumulation (including elevation) Vector to raster 100 m resolution	
Proximity	Distance to estuaries https://datasets.seed.nsw.gov.au/dataset/estuaries12439	Distance accumulation (including elevation) 100 m resolution	Estuary hydro regimes influence inundation of coastal wetlands (Felipe Bairos Moreira et al. 2015)
	Distance to waterbodies https://knowledge.dea.ga.gov.au/data/product/dea‐waterbodies‐landsat/	Distance accumulation (including elevation) Shapefiles between 1987 and 2022, > 80% occurrence suggesting permanent waterbodies 100 m resolution	Critical for amphibian habitat and breeding (Blachnik et al. 2019; Knapp et al. 2003; Mathwin et al. 2023)
	Distance to coastline	Distance accumulation (including elevation) Coastline was clipped to New South Wales extent from Australia 100 m resolution	Affects amphibian growth (Lorrain‐Soligon et al. 2021, 2022)

Categorical layers (DEA Landcover, vegetation type and soil type) were resampled from the original resolution to 100 m using nearest neighbour to preserve class values (e.g., recoded as 1—Native Grassland); continuous layers (climate, proximities, slope) used bilinear interpolation (Table 1, Figure S3). Some of the models utilised in this study require complete rasters, so NoData cells and areas within building footprints were assigned as zero (unsuitable habitat). The 100 m resolution minimised resampling (most layers native) while capturing fine‐scale habitat, improving model efficiency (Meineri and Hylander 2017; Nezer et al. 2017; Figure S4).

The lack of a public soil salinity raster layer prompted the creation of a custom layer from 14,000 TERN Soil Data Federator samples (New South Wales Natsoil/ASRIS), excluding pre‐1970 records, duplicates and outliers. Empirical Bayesian Kriging (EBK) in ArcGIS Pro interpolated these with New South Wales soil type maps, accounting for spatial autocorrelation via iterative model adjustment (Gharaibeh et al. 2024; Zaresefat et al. 2024). Salinity was expressed as electroconductivity (dS/m).

### 2.4 Variable Selection and Multicollinearity Screening

Seven predictor variables were selected for species distribution model analysis from 20 candidates (Table 1): static variables (proximity to coast and water, slope, soil salinity, native vegetation) and temporally dynamic variables (relative humidity at lowest temperature, DEA Landcover). To reduce the initial set of predictor variables, multicollinearity was assessed in R Studio (v2025.09.1.401; R Core Team 2025), using Spearman correlations (> 0.7) VIF and Cramer's V for categorical variables (> 5; (Geyle et al. 2025): Figures S2 and S3, Table S1). Climatic variables and proximities (to estuaries, coast and also elevation) showed high collinearity. To resolve this prior to the final species distribution model (ensemble) being run, MaxEnt, Random Forest (RF) and Boosted Regression Trees (BRT) were iteratively run via *SDMtune* (Vignali et al. 2020), swapping each of the eight correlated variables while holding non‐correlated factors constant. This resulted in the delineation of which of the correlated variables (highly correlated with each other) could be included in the ensemble.

### 2.5 Species Distribution Modelling

Species distribution models were fit to the filtered occurrence records from the full‐period and contemporary period. MaxEnt (*dismo* and *rJava* packages) (Phillips et al. 2006; Phillips and Schapire 2004) was parameterised with linear, quadratic, product and hinge features (betamultiplier = 1, threshold features disabled, cloglog output); these feature classes allow for both straightforward and more complex responses of predicted habitat suitability to environmental variables. Random Forest (RF) (*randomForest package*) [1000 trees] (Breiman 2001) and Boosted Regression Trees (BRT) (*gbm* package) [tree complexity = 5, learning rate = 0.01, bag friction = 0.7] (Elith et al. 2008) followed methods from Geyle et al. (2025) and Von Takach et al. (2020).

For each period, 10,000 randomly distributed background points were generated within the IBRA raster extent. Environmental values were extracted and continuous variables standardised using pooled means/SDs across periods for comparability. Five‐fold spatial block cross‐validation (*blockCV* package) evaluated performance via AUC, with Youden's J thresholds derived from pooled out‐of‐fold cross‐validation predictions following methods from Geyle et al. (2025). AUC‐weighted ensemble suitability maps were generated for both time periods; habitat suitability change was computed via pixel differences and classified as moderate (0.05–0.20) or large (≥ 0.20) in either direction, with thresholds set relative to observed range changes across the study area. Partial dependence plots (PDPs) assessed variable responses across models and periods.

### 2.6 Hypervolume Niche Analysis

The top four species distribution model predictors (proximity to coast and water, soil salinity, relative humidity minimum at lowest temperature, Figure S5) were pooled, *z*‐score standardised (mean/SD across periods), and separated into full‐period (*n* = 1000 replicates) and contemporary (*n* = 200 replicates) occurrence datasets, following Scheele et al. (2023) and Mammola and Cardoso (2020) to control computation time and convergence. Four‐dimensional niche hypervolumes were estimated using one‐class Support vector machine (v = 0.01, y = 0.5; hypervolume package v3.1.6; Mammola and Cardoso 2020). Subsampling without replacement reduced period‐specific biases. Niche overlap (Jaccard, Sorensen), volume differences (Wilcoxon rank‐sum) and β‐diversity were quantified via *BAT package*; centroid shifts assessed niche displacement. Visualisation used Principal component analysis (PC1/PC2; 500 random points/period; chull convex hulls) with variable loadings and period centroids.

### 2.7 Inundation Projection

Potential future inundation for sea level rise was estimated by using an elevation raster derived from tiled LiDAR‐derived DEM 5 m (margin of error ±0.3 m), with gaps filled with 1 m LiDAR‐derived tiles and a minimum elevation value set to 0 m Australian Height Datum (AHD; New South Wales Government, ELVIS) to determine inundation of the species' critical habitat when overlayed with inundation scenarios (tidal inundation, 0.5 m AHD, 1 m AHD, 1.5 m AHD). For habitat suitability, *critical habitat* was defined from the continuous ensemble suitability surface as areas exceeding the 10th percentile of suitability values extracted at known presence locations (contemporary > 0.77), ensuring 90% of known occurrence records were retained within classified habitat, following similar protocol discussed in Freeman and Moisen (2008).

## 3 Results

### 3.1 Extent of Occurrence

The buffer analysis conducted in ArcGIS Pro 3.5.0 found that 86% of all records from the full period were found within 1 km of the coastline (including large coastal bodies, e.g., Lake Macquarie in New South Wales). Alpha‐hull analysis (α = 8) demonstrated a 76% contraction in the extent of occurrence from full period (1970–2024; 34,092 km^2^) to contemporary (2000–2024; 8298 km^2^) time period (Figure 2) for *R. aurea* . The contemporary hull was 99.94% nested within the full‐period hull, indicating contraction without novel occupancy. Spatial overlap was low (Jaccard = 0.21, Sorensen = 0.34), confirming severe range loss and fragmentation.

**FIGURE 2:**
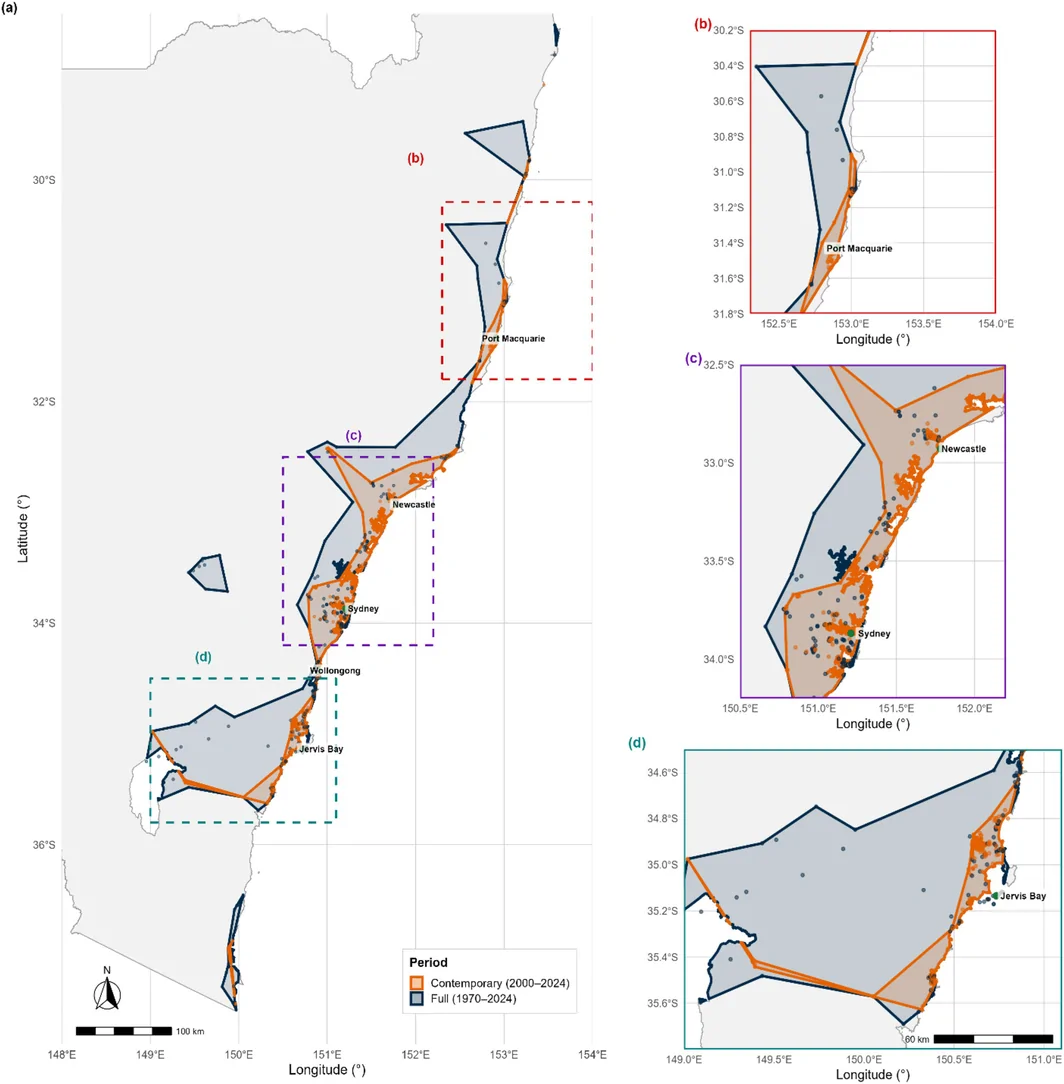
(a) Full‐period distribution extent (blue) and contemporary distribution extent (orange) displaying changes in alpha hulls for the full coastline of New South Wales, (b) zoomed in on northern section of coastline, (c) zoomed in on middle section of coastline and (d) zoomed in on southern section of coastline.

### 3.2 Species Distribution Models

Models showed excellent discrimination (AUC = 0.97–0.98, TSS = 0.85–0.89, sensitivity = 0.90–0.97, specificity = 0.90–0.94, Boyce Index (predictability) = RF, 0.74–0.78, MaxEnt 0.40–0.52). Final VIF scores (< 2.36) confirmed variable independence. RF (1000 trees), BRT (tree complexity = 5, learning rate = 0.01) and MaxEnt achieved high performance (AUC 0.96–0.98), with equal weighting (~33% per model). MaxEnt performed best for full period data (AUC = 0.97) and RF for contemporary period data (AUC = 0.98).

Proximity to the coast was the dominant influence in all models (13%–63% VI, Figure 3), with minimum relative humidity at lowest temperature having peak suitability for the species at 91%–92%, reflecting coastal conditions. A 96% habitat suitability loss occurred between the historical and contemporary ensemble rasters (defined by pixel change), comprising 82.2% moderate negative change (5%–20% loss of suitable habitat) and 14.5% large negative change (≥ 20% loss of suitable habitat) (Figure 6; Table 2). Greater Sydney exhibited strong inland declines (up to 60%), while Northern Rivers coastal areas showed ~30% suitability gains (Figure 4).

**FIGURE 3:**
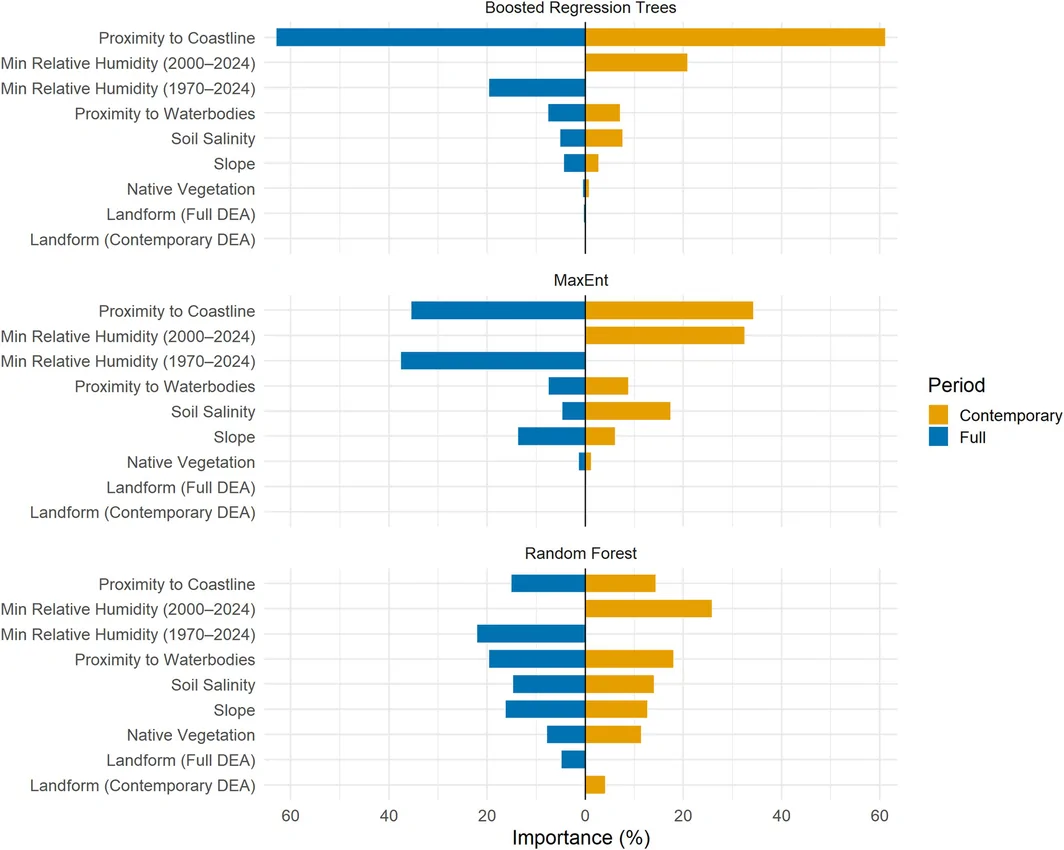
Variable importance (%) of environmental predictors for *Ranoidea aurea* across three species distribution modelling algorithms (BRT, RF, MaxEnt). Variable importance values were standardised within models to enable comparison among algorithms and time periods, highlighting the consistent influence of proximity to coastline and lower and more model‐dependent contributions of minimum relative humidity and soil salinity.

**TABLE 2:** Changes in habitat suitability between full and contemporary ensemble projections for *Ranoidea aurea* .

Habitat suitability	Area (km^2^)	Percentage (%)	Combined %
Large decrease (20%>)	3495.5	14.53	96.76
Moderate decrease (5%–20%)	19776.2	82.23	
Moderate increase (5%–20%)	727.2	3.02	3.23
Large increase (20%>)	51.47	0.21	

*Note:* Cells show the area (km^2^) and percentage of the study region comprising four categories of change: Large Decrease (≥ 20% loss), Moderate Decrease (5%–20% loss) (red cells), Moderate Increase (5%–20% gain) and large increase (≥ 20% gain) (green cells). Values represent pixels with a change in ensemble habitat suitability of at least 0.05 (5%), with smaller changes excluded. About 96% of the area with ≥ 5% change shows decreased suitability, demonstrating a strong, range‐wide suitability loss.

**FIGURE 4:**
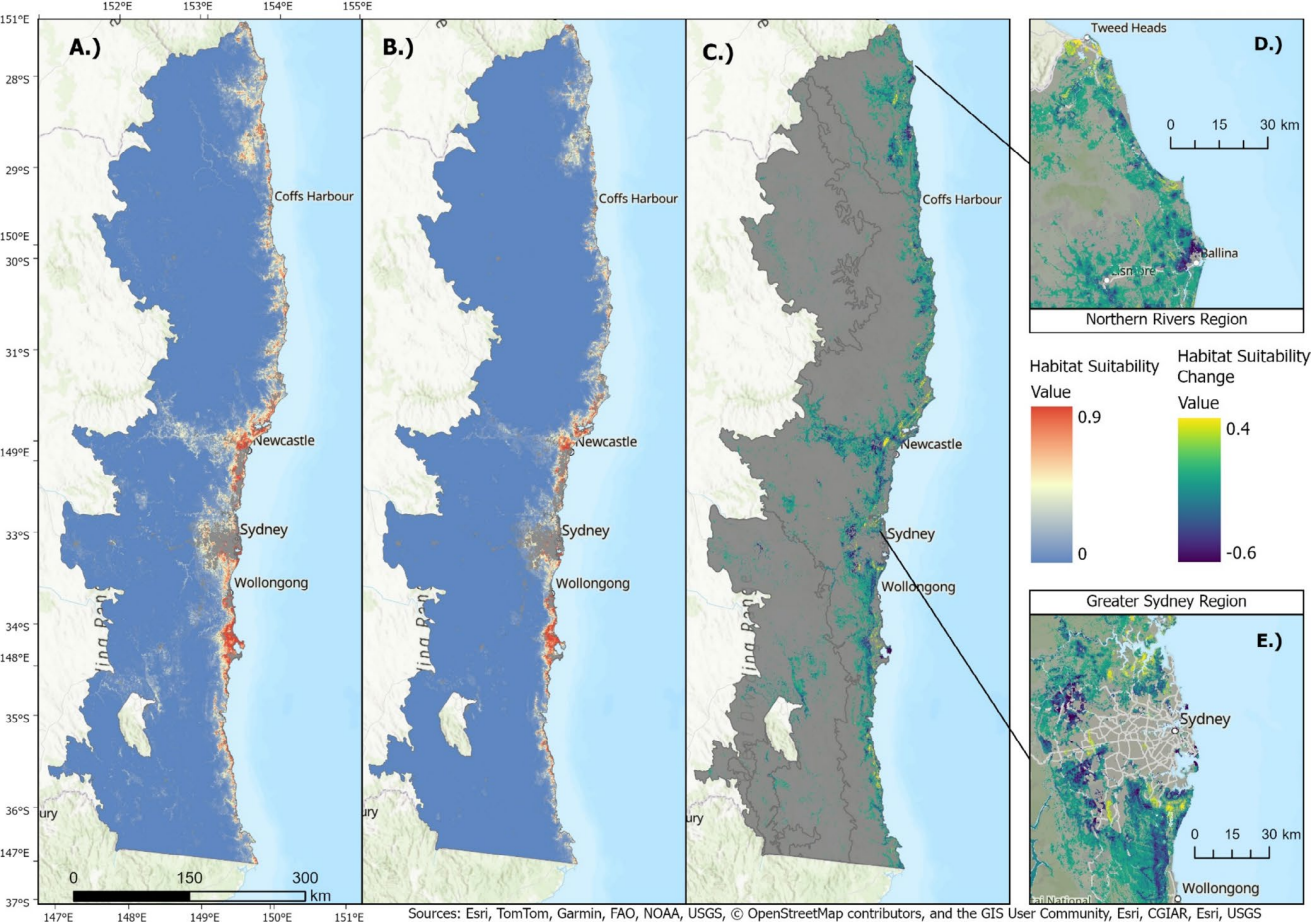
Ensemble habitat suitability for *Ranoidea aurea* and changes between full and contemporary periods. Panels (A) and (B) show ensemble habitat suitability for the full (1970–2024) and contemporary (2000–2024) periods respectively. Grey represents the study area and masks out building footprints (excluded data). Panel (C) shows the change in habitat suitability between periods. Inset (D) shows the Northern Rivers Region with some positive habitat suitability change towards the north of the state. Inset (E) shows largely negative change for the outer Sydney region.

### 3.3 Hypervolume Niche Analysis

Hypervolume analysis (one‐class Support vector machine, 200/1000 replicates) showed 57% niche volume contraction (full median: 47.3; contemporary: 20.1; Wilcoxon *p* < 0.001; Figure 5). Jaccard (0.359) and Sorensen (0.528) indices indicated moderate niche overlap, while β‐diversity partitioning showed high turnover (high β_total and β_replacement, and low β_richness) (Figure 5), confirming environmental filtering.

**FIGURE 5:**
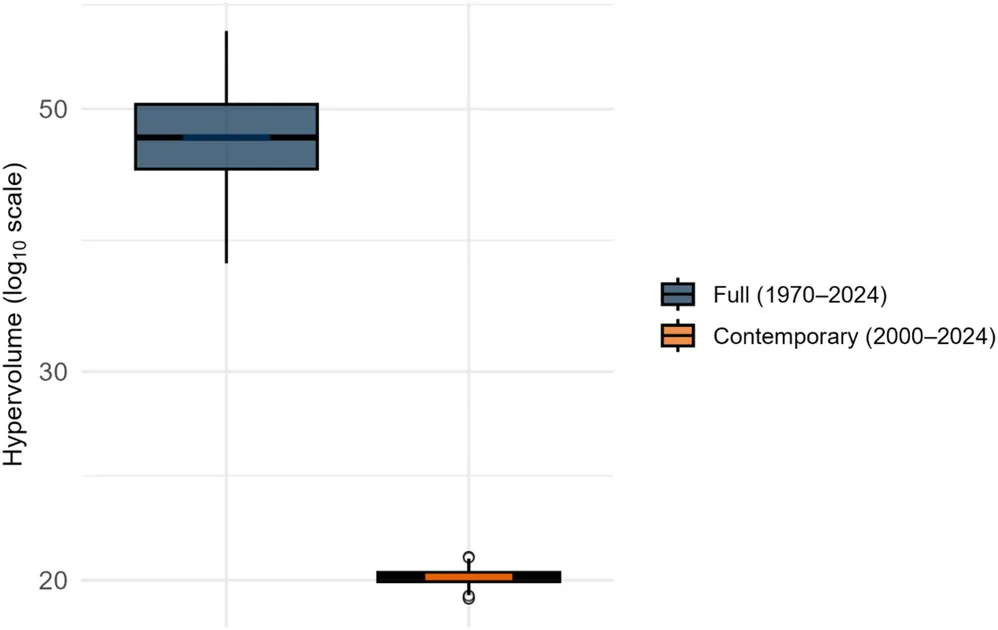
Comparison of hypervolume niche sizes for *R. aurea* between full‐period (1970–2024) in blue and contemporary (2000–2024) period in orange. Boxplots represent the distribution of hypervolume sizes across replicate calculations. Larger values indicate broader niches encompassing a wider range of environmental conditions, whereas smaller values reflect narrower niches. Outliers are represented by dots.

The full‐period niche centroid was located at PC1 = 0.049, PC2 = 0.002, while the contemporary niche centroid shifted to PC1 = −0.096, PC2 = 0.008 (Figure 6). All environmental shifts were significant (*p* < 0.05): negative for relative humidity at minimum temperature (−0.43) (towards less humid conditions) and negative (showing closer proximity to variable) for proximity to the coast areas, waterbodies (−0.52, −0.16) and positive for soil salinity (+0.03).

**FIGURE 6:**
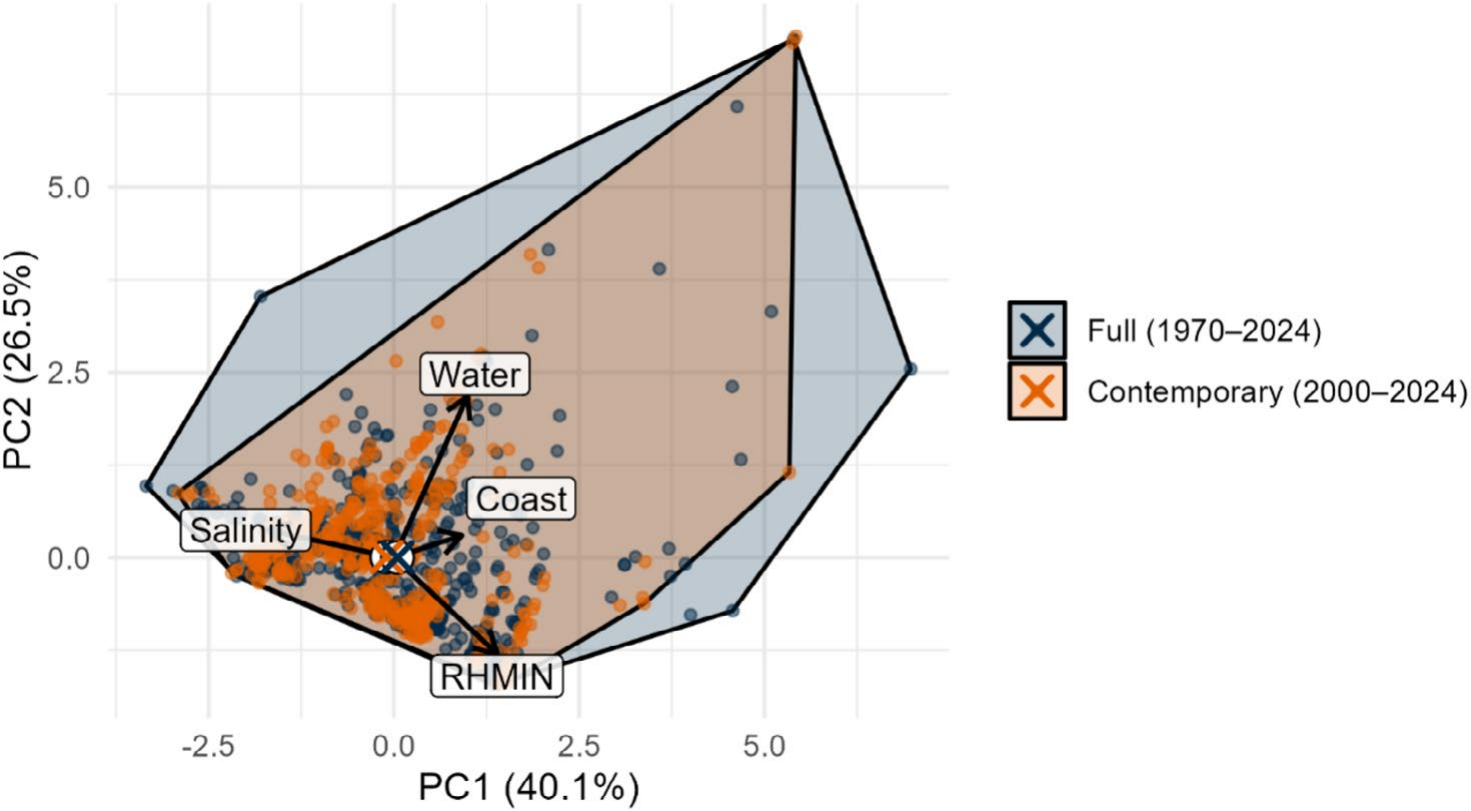
Principal component analysis of the environmental niche of *Ranoidea aurea* . Principal components (PC1, PC2) represent weighted combinations of the four key predictors (relative humidity at minimum temperature (RHMIN), proximity to coast (Coast) and water (Water) and Soil salinity (Salinity)) summarising the main environmental gradients used by the species. Points are occurrence records plotted in reduced environmental space; the centroid (X) marks the average niche position for each period, demonstrating a shift towards closer proximity to the coast and waterbodies, and higher salinity. Arrows show the direction and relative strength of each environmental variable in the principal component analysis space, with arrow direction indicating the gradient of increasing variable values.

### 3.4 Inundation

Under the projected inundation scenarios, the total area of critical habitat was compared against the area inundated under each scenario (binary 0, 1). The results show that 4.4% of critical habitat is inundated under 0.5 m AHD, 12.5% under 1 m AHD and 20.5% under 1.5 m AHD.

## 4 Discussion

Geometric, correlative and niche‐based analysis provided strong and consistent evidence of *Ranoidea aurea* now existing in a narrower zone than its original environmental envelope, due to a directional niche contraction into saline, coastal refugia (Mahony et al. 2013). However, inundation modelling showed that this niche is now threatened by climate change‐driven sea level rise. The species is currently highly dependent on the scattered habitats still available, with much of the coast already impacted by intense coastal development (Stocker et al. 2025). This double‐jeopardy refugial squeeze threat is critical to consider in managing the biodiversity crisis, as many species are likely to face similar challenges (Mainka and Howard 2010). Our results highlight that using a combination model analysis provides strong evidence of contraction, its direction and drivers. In the case of *R. aurea* , habitat suitability has altered, with only a small proportion of the original niche still used by contemporary populations, as well as 4.4%–20.5% of its critical habitat likely to become unsuitable under inundation based sea‐level rise scenarios.

### 4.1 Proximity to Coastline and Soil Salinity as Key Predictors

*Ranoidea aurea* exhibited a spatial range contraction without any substantial shift into novel habitat, evidenced by 76% loss in Extent of Occurrence with 99.94% nesting of the contemporary period hull within the full‐period hull. The hypervolume analysis demonstrated a reduction in niche volume (57%) as well as a shift towards coastal regions and regions with higher salinity, consistent with proximity to the coastline being the most predictive variable in current species distribution suitability, and soil salinity having a moderate impact. What these findings reveal is that the species is not being squeezed inwards into pockets of habitat across its range, but rather, squeezed directionally towards the coast. Similar squeezes are apparent in alpine specialists being forced to higher altitude, driven by climate change (Girardello et al. 2010; Tiberti et al. 2021).

*Ranoidea aurea* is commonly found in coastal wetlands, which fluctuate between saline and freshwater environments. Stockwell et al. (2012, 2015) hypothesises that this species' connection to the coastline could relate to salt's antifungal properties, which reduce the spread and infection rate of chytrid fungus. This finding suggests that *R. aurea* populations persist in coastal habitats due to potential periodic salt influxes that occur from coastal processes, creating less favourable conditions for the pathogen and increasing survival rates in *R. aurea* populations (Clulow et al. 2018; Stockwell et al. 2015). Other climatic factors such as temperature and humidity may also influence the infection rate of the pathogen, with coastal environments often hosting higher temperatures compared to habitats further inland (Pepler 2011).

These low‐lying coastal areas are likely to be refuges for *R. aurea* from chytrid due to the higher salinity inhibiting the fungus, thus creating a zone where the niche of the threatened and threatening species do not strongly overlap (Callen et al. 2023; Stockwell et al. 2015). Yet these refugia are now vulnerable to human‐driven climate change, which is causing sea level rise, changing the frequency and intensity of saltwater inundation (Doody 2004; Pontee 2013), which the species is only partially tolerant to. This wedges the species between two spatially opposed threatening processes, in effect creating a highly spatially constricted zone of functional habitat where both threats are sufficiently low to allow for population viability (e.g., Goldilocks zone).

Existing research exploring amphibian proximity to coastal habitats focuses primarily on morphology, movement and breeding with conclusions suggesting that salt exposure due to landward sea‐spray and deposition at breeding sites is a driving factor for population structures, especially growth rates (Barratt et al. 2017; Cline and Hunter 2016; Lorrain‐Soligon et al. 2022, 2024; Hopkins et al. 2016). Furthermore, salinity also acts as an oviposition cue for breeding in some frog species (Haramura 2007). However, these studies did not explore coastal salinity in the context of chytrid, offering an overlooked mechanism influencing amphibian occupancy and survival, especially species heavily affected by disease such as *R. aurea* (Mahony et al. 2013; Stockwell et al. 2010).

Many benefits exist between the exchange of salinity in wetlands and overall survival of anuran species since these conditions are often not tolerable to many individuals, resulting in potential release from interspecies competition and predation (Hopkins and Brodie 2015). These coastal systems, however, are increasingly threatened by coastal squeeze from developments and human‐induced climate change (Doody 2004; Pontee 2013), with factors such as habitat destruction and alteration, erosion, decline in water quality, climatic variation and intensity of storms noted as current stressors (He and Silliman 2019). An emerging concern for coastal systems is sea level rise, which can result in hypersaline environments (Hallett et al. 2017; Saintilan et al. 2019). Although salinity has its benefits for amphibians, if it reaches critical thresholds for frog species, especially at water dependent life stages (potentially 10‐16% seawater for *R. aurea*), it can negatively affect survival, growth and development (Chinathamby et al. 2006; Christy and Dickman 2002; Smith et al. 2007) and could ultimately lead to extinction through reduction of available breeding habitat. Managing coastal habitats will thus be critically important for the survival of amphibian species occupying the coast.

### 4.2 A Major Niche Contraction Potentially Driven by Humidity

Niche contraction in amphibians has been demonstrated globally, reflecting a pattern established by the taxa experiencing widespread population declines. Scheele et al. (2023) found that chytrid fungus resulted in directionally characterised niche contractions and centroid shifts in Australian frog species affected by the disease into lower elevation areas with higher temperatures and precipitation. Similar findings were documented by Granados‐Martínez et al. (2021) with niche and range contractions caused by the disease, also showing a shift towards warmer refugia.

We found that *R. aurea* has experienced a large contraction (57%) in its environmental niche and a significant negative change in suitable habitat (96%). There is moderate overlap between the two environmental niches, suggesting contraction as well as environmental displacement. These results further emphasise that the species now only occupies a narrow environmental envelope, posing a threat to its conservation and viability. Past literature exploring the implications of niche changes in amphibians has noted a similar trend in relation to *R. aurea* and *R. raniformis* , with Scheele et al. (2023) and Heard et al. (2024) previously detecting species decline and contraction, with predominately mild and wet climatic conditions, as well as low elevation as drivers.

In this study, relative humidity (RH) at minimum temperature was the top climatic driver for the species, with partial dependence plots showing peak habitat suitability around 91%–92% RH during minimum temperature. The centroid shift (between periods) towards lower RH values at minimum temperature indicates movement to slightly lower humidity conditions; however, this is still within a coastal zone that represents high absolute humidity. The observed shift towards lower RH at minimum temperature in coastal habitats may reflect a broader climatic trend, with land surface warming faster than adjacent oceans, causing reduced RH on land despite stable or increasing moisture (Byrne and O'Gorman 2018). Along the New South Wales coastline, warmer ocean conditions generate more humid and warm nights, especially during summer, compared to inland localities (Pepler 2011). Since the remaining populations of *R. aurea* are within this coastal zone, it is important to consider that the species is still utilising a high humidity zone; further, regionally coastal areas contain a significantly higher RH than their inland counterparts. This result indicates subtle potential climatic changes within a highly favourable niche condition, which could lead to effects on chytrid dynamics and suitable habitat conditions.

Scheele's study (Scheele et al. 2023) found that niche centroids of frog species affected by chytrid fungus contract towards areas of low elevation, warm temperatures and higher precipitation. This shift into warmer climates is supported by other literature suggesting that optimum growth of the fungus occurs between 17 and 26 degrees Celsius and with continual presence of water (Gajewski et al. 2021; Rohr et al. 2011; Turner et al. 2021). Since chytrid predominantly thrives in mild and wet climates, its presence alone does not fully explain why *R. aurea* is mostly restricted to coastal zones with potentially multiple overlapping pressures at play. These threats can lead to possible trade‐offs or ecological filtering occurring, with suitable breeding sites, food resources, habitat availability, exposure to disease and invasive species collectively narrowing the range of optimal environments. These threats could cause further squeezing of the species into small, isolated pockets, which itself could explain their fragmented extent in addition to the fragmentation caused by past human development and urbanisation. Further to this, in some coastal habitats, high recruitment may offset reduced adult survival rates, with areas where recruitment cannot offset losses, resulting in likely extinction (Heard et al. 2024; Valenzuela‐Sánchez et al. 2021). On a local scale, altered hydrological regimes, predation from the invasive mosquito fish ( *Gambusia holbrooki* ) and food availability potentially contribute further to existing large‐scale stressors and their repercussions (Hamer 2002; Klop‐Toker et al. 2018; Morgan and Buttemer 1996).

### 4.3 A Significant Reduction in Habitat Suitability, With Regional Scale Differences Providing Insights Into Conservation

Significant negative shifts were observed in predicted habitat suitability for *R. aurea* , with the model suggesting up to 14% large negative change and 82% moderate negative change, with limited positive gains noted (3.9% and 0.2%) between full‐period and contemporary ensemble models. This means that up to 96% of the species' known habitat has seen a negative decrease in suitability, suggesting a loss of habitat quality across almost the entirety of its distribution. This matches the output of hypervolume and extent of occupancy analysis, whereby the species range and niche have contracted, particularly at its edges (inland) and also occupies fewer and more unsuitable environmental conditions. This suggests a shift in key environmental parameters, with the species now confined to the remaining coastal refugia, in which it is facing pressures from coastal squeeze and human‐induced climate change.

The ensemble model provides a cohesive result across multiple models, which suggests urgency in planning the conservation of these remaining coastal populations and associated habitats. Specifically, locations inland of the greater Sydney region saw significant negative change in habitat suitability, which reflects the current declining trend of populations in this region (West et al. 2020). While some northern coastal regions saw a positive increase in predicted habitat suitability, others suggested a negative trend, which could indicate a narrowing of ideal habitats due to climatic pressures such as drought leading to a decrease in rainfall (Goldingay and Newell 2005). A positive aspect of these northern coastal regions is that they are situated in zones (e.g., National Parks) with significantly lower impacts from urbanisation, development (the similar *coastal squeeze* process) and pollution factors. Additionally, they have higher temperature conditions compared to the southern New South Wales coastal site populations (Goldingay et al. 2021), which could potentially prevent the infection and spread of chytrid.

Proximity to the coastline was the most important variable for predicting distribution of the species and should be further investigated to understand what factors, aside from salinity and relative humidity, make a closer proximity to the coastline more idealistic for *R. aurea* . Species distribution models do not commonly use distance‐based parameters, with climatic and topographic parameters often highlighted as the most influential (Cruz‐Cárdenas et al. 2014; Jiménez‐Valverde et al. 2008). However, in the context of the target species, research has highlighted the role of salinity in limiting chytrid fungus and points to a potential coastal shift in the species' distribution (Callen et al. 2023; Klop‐Toker et al. 2017; Stockwell et al. 2012, 2015), making distance‐to‐coastline an important parameter within this study. This highlights the importance of using variables that are significant to the ecology of target species as well as producing models that are taxa appropriate (Kearney and Porter 2009). Another common theme throughout *R. aurea* populations is that these coastal environments contain wetlands, lakes and lagoons. These populated coastal zones systems are often heavily hydrologically altered to manage flooding of commercial and residential areas (Kennish 2002). This has significant strain on the natural hydrology of the system and can have long‐term implications for the refugia habitat and overall conservation of the species.

### 4.4 Limitations

#### 4.4.1 Survey Accessibility and Bias

Accessibility bias towards public land (e.g., Parks, Crownland, Indigenous Land Councils) possibly inflates coastal occurrence records, with private lands often lacking occurrence records due to instances of inaccessibility. This limitation is commonly noted in other species distribution model papers (Kramer‐Schadt et al. 2013; Lissovsky and Dudov 2021), however, historical locations of *R. aurea* have been extensive and repeatedly surveyed rendering this bias unlikely.

Survey and sample bias may partially influence the apparent strength of coastal refugia identified in our models (e.g., Tweed Heads). Occurrence records are spatially clustered around major population centres and long‐term monitoring sites, potentially leading to overstating habitat suitability relative to under sampled areas. However, to our knowledge, there is no clearly documented, long‐running *Ranoidea aurea* monitoring program in the New South Wales North Coast Region (Tweed Heads). Since our analysis uses presence‐only data, the absence of the species cannot be truly known, so contraction may be conflated with undersampling, particularly regarding inland sites.

A limitation was noted for the alpha hull analysis, in which a large alpha hull was selected (α = 8) to include isolated occurrences that would have been otherwise excluded from the analysis. This results in a potential overestimate of distribution, representing a compromise of distribution rather than an optimal fit of the species hull. With consistency across alpha hulls, species distribution models and hypervolume analysis of a contraction towards a narrow coastal band suggests that the broad pattern of a chytrid‐mediated coastal refugial niche exists, even in the presence of these potential sampling and analysis limitations.

#### 4.4.2 Environmental Layers

Key spatial dataset limitations exist in the DEA landcover, soil salinity, native vegetation and proximity to waterbodies layers. The DEA landcover was taken from yearly grids, with the mode extracted for each period 1988 (earliest date)–1999 and 2000–2024, meaning 1970–1987 was unaccounted for. Soil salinity data existed from 1970 to 2022, excluding 2023–24. The native vegetation layer is aggregated between 2008 and 2017 and the proximity to waterbody layer (maximum wet extent since 1986) results in these parameters better representing the conditions within the contemporary period compared to the full‐period.

## 5 Conclusions

Our study used integrated multi‐modelling approaches as a proof of concept of the refugial squeeze phenomena that is likely to affect species that face multiple, yet opposing stressors. This is expected to become more relevant as climate envelopes shift due to rising temperatures and erratic weather patterns. In coastal zones, this refugial squeeze is demonstrated by urbanisation and sea level rise predictions. Our model results showed that the coastally constrained threatened amphibian, *Ranoidea aurea* has undergone a significant contraction in both its range and niche composition since the introduction of *Batrachochytrium dendrobatidis*, with alpha hull, hypervolume and species distribution model analysis now quantifying the previously observed strong association with the New South Wales coastline. This finding further strengthens the critical importance of coastal habitats to disease‐susceptible amphibians, but sea‐level rise predictions now jeopardise this refugia for the species. This study also highlights the importance of exploring multiple concepts for niche changes and parameter influences on distributions in an ecological and geographical context, confirming that not all coastal areas are equal in terms of habitat suitability.

## Author Contributions

**Kate Tunstill:** conceptualization (lead), data curation (lead), formal analysis (lead), funding acquisition (lead), investigation (lead), methodology (lead), validation (lead), visualization (lead), writing – original draft (lead), writing – review and editing (lead). **Matt W. Hayward:** conceptualization (lead), funding acquisition (supporting), project administration (supporting), supervision (supporting), validation (supporting), visualization (lead), writing – original draft (lead), writing – review and editing (lead). **Hannah Power:** conceptualization (lead), funding acquisition (supporting), project administration (supporting), supervision (supporting), validation (supporting), visualization (lead), writing – original draft (lead), writing – review and editing (lead). **Darren Southwell:** conceptualization (supporting), data curation (supporting), formal analysis (supporting), methodology (supporting), validation (supporting), visualization (supporting), writing – original draft (supporting), writing – review and editing (supporting). **John Gould:** conceptualization (supporting), methodology (supporting), writing – review and editing (supporting). **Will Farebrother:** data curation (supporting), formal analysis (supporting), methodology (supporting), software (supporting), writing – review and editing (supporting). **Michael Mahony:** writing – review and editing (supporting). **Alex Callen:** conceptualization (lead), funding acquisition (lead), project administration (lead), supervision (lead), validation (lead), visualization (lead), writing – original draft (lead), writing – review and editing (lead).

## Conflicts of Interest

The authors declare no conflicts of interest.

## Supporting information


**Figure S1:** All alpha hulls (a = 2, 4, 6, 8, 10) for EOO fitted against occurrence records (full and contemporary) to determine which best represents the extent of *R. aurea* with complete hulls.
**Figure S2:** Heatmaps of Pearsons correlation analysis (rho scores) between original environmental variables, full period correlation (A) and contemporary period correlation (B) between variables.
**Figure S3:** Cramer's V heatmap showing correlation between categorical variables.
**Figure S4:** Flowchart of steps involved with manipulating spatial data, resulting in final input into R studio.
**Figure S5:** Four key predictor for species distribution and niche change, proximity to waterbodies (metres) (A), Soil salinity (dS/m) (B), proximity to coast (metres) (C) and relative humidity at minimum temperature for the full period (%) (RHMIN) (D). Map created using ArcGIS Pro 3.5.0 (Esri 2025), Map projected in GDA2020/NSW Lambert (EPSG:8058); graticule displayed in geographic coordinates (GDA2020).
**Table S1:** VIF values over 5 for contemporary environmental parameters.

## Data Availability

All data and code used in this study is available in the Data Repository Zenodo: doi: 10.5281/zenodo.20077839.
