# Supplementary material for: The Refugial Squeeze: A Globally Relevant Mechanism of Species Decline Revealed by a Threatened Amphibian
Source: Ecol Evol. 2026 Oct 15;16(10):e74417. doi: 10.1002/ece3.74417 (PMC13671101; doi:10.1002/ece3.74417)
Supplement: Supplementary file 1 [file ECE3-16-e74417-s001.docx]

Figure S1 – All alpha hulls (a = 2, 4, 6, 8, 10) for EOO fitted against occurrence records (full and contemporary) to determine which best represents the extent of *R. aurea* with complete hulls.

A

B

Figure S2 Heatmaps of Pearsons correlation analysis (rho scores) between original environmental variables, full period correlation (A) and contemporary period correlation (B) between variables.

Table S1 VIF values over 5 for contemporary environmental parameters.

| **Variable** | **VIF CONTEMPORARY** | **VIF FULL** |
| --- | --- | --- |
| RH MIN | 8.00 | 4.62 |
| ELEVATION | 9.59 | 7.14 |
| EVAP | 15.41 | 11.52 |
| RH MAX | 26.65 | 15.02 |
| DIS ESTUARIES | 57.04 | 52.97 |
| DIS COAST | 68.10 | 59.97 |
| MAX TEMP | 160.71 | 107.48 |
| MIN TEMP | 179.62 | 124.29 |

Figure S3 - Cramer's V heatmap showing correlation between categorical variables.

Figure S4 Flowchart of steps involved with manipulating spatial data, resulting in final input into R studio.

Figure S5 – Four key predictor for species distribution and niche change, proximity to waterbodies (metres) (A), Soil salinity (dS/m) (B), proximity to coast (metres) (C) and relative humidity at minimum temperature for the full period (%) (RHMIN) (D). Map created using ArcGIS Pro 3.5.0 (Esri, 2025), Map projected in GDA2020 / NSW Lambert (EPSG:8058); graticule displayed in geographic coordinates (GDA2020).
